# Pancreatic Damage in Ovarian Cancer–Associated Cachexia Is Driven by Activin A Signalling

**DOI:** 10.1002/jcsm.70096

**Published:** 2025-10-10

**Authors:** Amirhossein Abazarikia, Wonmi So, Yi Luan, Chandramohan Kattamuri, Thomas B. Thompson, So‐Youn Kim

**Affiliations:** ^1^ Olson Center for Women's Health, Department of Obstetrics and Gynecology, College of Medicine University of Nebraska Medical Center Omaha Nebraska USA; ^2^ Department of Molecular and Cellular Biosciences, College of Medicine University of Cincinnati Cincinnati Ohio USA; ^3^ Fred and Pamela Buffett Cancer Center University of Nebraska Medical Center Omaha Nebraska USA

**Keywords:** acinar cell atrophy, activin A, amylase, cachexia, follistatin 288

## Abstract

**Background:**

Cancer‐associated cachexia (CAC) is a severe metabolic disorder characterized by involuntary weight loss, skeletal muscle atrophy and adipose tissue depletion. It is a major contributor to morbidity and mortality in the advanced stages of various cancers. However, the impact of CAC on the pancreas remains largely unexplored.

**Methods:**

We used mice with constitutively active *PI3K* in oocytes, generated through a Cre‐inducible Pik3ca* knock‐in allele driven by *Gdf9*‐icre and performed histological and molecular analyses of the pancreas during cachexia development. Additionally, we examined pancreatic changes following ovariectomy and administration of Follistatin 288 (FST288).

**Results:**

Mice that developed cachexia symptoms associated with granulosa cell tumour (GCT) growth exhibited significant pancreatic atrophy compared to controls (Cre+ vs. Cre− at PD83, *p* < 0.0001), including reduced size of individual acinar cells (102.99 ± 12.19 μm^2^ vs. 207.94 ± 24.85 μm^2^ at PD83, *p* < 0.0001) and acinar units (346.41 ± 169.22 μm^2^ vs. 1193.59 ± 136.01 μm^2^ at PD83, *p* < 0.0001), despite comparable food intake between groups. Acinar cells exhibited a decrease in zymogen granules, reduced amylase expression and diminished amylase activity in both serum (0.29 ± 0.08 vs. 1.41 ± 0.40, *p* < 0.001) and tissue (0.37 ± 0.14 vs. 1.05 ± 0.29, *p* < 0.01). In contrast, pancreatic islets remained intact, as evidenced by histological analysis and preserved insulin expression. The pancreas of PD83 Cre+ mice also developed fibrosis and acinar cell death, characterized by elevated expression of collagen IV and α‐SMA, and TUNEL‐positive signals in acinar cells, respectively. Ovariectomy preserved body weight (2.66 ± 1.30 g for Cre+/OVX vs. 1.60 ± 0.97 g for Cre−) compared to Cre+ mice (−3.66 g) and maintained pancreatic function, suggesting that tumour‐derived factors from GCT contribute to the severity of cachexia. Acinar cells showed high expression of ACVR2B, leading to activation of downstream p‐SMAD3 signalling. Accordingly, activin A directly induced acinar cell atrophy in both ex vivo cultured pancreas (79.27 ± 19.03 μm^2^ vs. 171.14 ± 27.01 μm^2^, *p* < 0.0001) and 266‐6 acinar cells, as evidenced by reduced acinar cell size and decreased amylase production. Injection of FST288, an activin A inhibitor, rescued pancreatic acinar atrophy (252.95 ± 11.59 μm^2^ in Cre+/FST288 vs. 97.25 ± 12.37 μm^2^ in Cre+, *p* < 0.001) without affecting GCT tumour size. Ex vivo culture of pancreas and 266‐6 acinar cells exposed to activin A confirmed that activin A directly induces pancreatic damage.

**Conclusions:**

These findings demonstrate pancreatic damage occurs during CAC development and highlight the critical role of activin A in this process. Targeting activin A signalling may represent a promising therapeutic strategy to mitigate cachexia in cancer patients and preserve pancreatic function.

## 1 Introduction

CAC is a complex, multifactorial metabolic disorder [1, 2] and a major contributor to morbidity and mortality in the advanced stages of various cancers, including pancreatic, gastric, oesophageal, lung, colorectal, liver, head and neck, ovarian, leukaemia, lymphoma and sarcoma [3, 4, 5, 6]. Characterized by involuntary weight loss, CAC leads to severe frailty due to skeletal muscle atrophy, adipose tissue depletion and organ dysfunction [7, 8]. This condition not only deteriorates patients' quality of life but also reduces chemotherapy efficacy while increasing its toxicity, ultimately contributing to higher mortality rates [4].

Although skeletal muscle and adipose tissue wasting are well‐established hallmarks of CACs [1, 9, 10], recent studies have emphasized both local and systemic disruptions, including elevated resting energy expenditure, mitochondrial dysfunction, gut microbiome alterations and inflammation‐induced anorexia, insulin resistance and excessive glucocorticoid release [11, 12, 13, 14, 15, 16, 17]. These factors drive a negative energy balance and anabolic resistance, primarily mediated by inflammatory cytokines, leading to widespread organ involvement [18, 19, 20, 21].

The pancreas plays a crucial role in metabolic homeostasis, with its endocrine function regulating glucose metabolism via the islets of Langerhans and its exocrine function producing digestive enzymes, such as amylase, for nutrient absorption [22, 23]. In pancreatic cancer, the pancreas undergoes progressive structural and functional deterioration, leading to pancreatic dysfunction, maldigestion, malabsorption and impaired insulin production. These changes exacerbate malnutrition, metabolic decline, muscle wasting and systemic deterioration, ultimately driving the development of pancreatic cancer‐driven cachexia [24, 25]. Consequently, pancreatic cancer is associated with poor surgical outcomes and extremely low survival rates [26, 27]. However, the direct impact of cachexia induced by other cancer types on an otherwise healthy pancreas remains unclear. Notably, dysfunction of acinar cells is known to contribute to exocrine pancreatic insufficiency (EPI) [28], increasing the risk of chronic pancreatitis, malnutrition and further metabolic dysregulation [29].

Despite CAC's profound impact on disease prognosis and quality of life, its effects on pancreatic function remain poorly understood. In this study, we utilized a genetically engineered mouse model of GCT with an oocyte‐specific Pik3ca* knock‐in [10, 30, 31]. GCTs are sex cord‐stromal tumours that constitute approximately 5% of human ovarian tumours and are associated with high recurrence and mortality rates. These mice, serving as mouse models of ovarian cancer, develop cachexia within 2 weeks of GCT formation, exhibiting body weight loss, adipose tissue loss, muscle atrophy, pancreatic damage and sudden death, accompanied by elevated serum activin A levels, a member of the TGF‐β superfamily [10, 31]. Furthermore, we demonstrated that the GCT is the source of activin A production and that activin A is a potential causal factor in the development of cachexia symptoms in this mouse model [10].

To investigate the effects of tumour growth and CAC progression on pancreatic morphology and function, we examined the pancreas before and after CAC onset. Additionally, we explored the molecular mechanisms driving pancreatic damage, focusing on the activin A signalling pathway. To assess its direct impact, we treated an acinar cell line with activin A and measured amylase and ACVR2B expression. Furthermore, we tested FST288, a natural activin A inhibitor [32], to determine its potential to mitigate pancreatic damage during CAC progression in a GCT mouse model.

This study highlights the underrecognized impact of CAC on pancreatic health and identifies activin A as a key mediator of pancreatic dysfunction. By demonstrating the protective effects of FST288, our findings provide new insights into potential therapeutic interventions aimed at preserving pancreatic function and improving metabolic outcomes in cachexia.

## 2 Materials and Methods

### 2.1 Animal

*Gdf9‐icre* mice (Stock Number 011062), which express Cre recombinase under the control of the mouse growth differentiation factor 9 (*Gdf9*) promoter, were purchased from the Jackson Laboratories. In these mice, Cre recombinase is specifically expressed in the oocytes of primordial follicles and is used to delete oocyte‐specific genes. Mice carrying a floxed allele for constitutively active *Pik3ca* (*C57BL/6‐Gt (ROSA)26Sor* ^tm7(Pik3ca*,‐EGFP)Rsky^ */J*, Strain Number 012343) were previously described [10, 30, 31]. This conditional allele, targeted to the *Gt (ROSA)26Sor* locus, contains a *loxP*‐flanked Neo‐STOP cassette that prevents transcription of the *Pik3ca* gene and EGFP. To generate transgenic mice with oocyte‐specific expression of constitutively active PI3K (Pik3ca*), homozygous Pik3ca* females were bred with heterozygous *Gdf9‐icre* ^*+*^ */* ^*−*^ males. These mice develop ovarian tumours, consistent with reports of other ovarian cancers harbouring *Pik3ca* mutations at a significant frequency. By PD65, mice typically present with large bilateral tumours without body weight loss, whereas body weight loss begins around PD70, and death generally occurs between PD80 and PD85. Therefore, PD65 and PD83 were selected as representative time points corresponding to the presence of large bilateral tumours without cachexia and to a cachectic state shortly before death, respectively. Genotypes were defined as Cre− (Gdf9‐icre−/−; Pik3ca*/w, wild type) or Cre+ (Gdf9‐icre+/−; Pik3ca*/w, conditional knock‐in). At sacrifice, weights of pancreas, skeletal muscles, adipose tissues, kidney and spleen were measured.

Ovariectomy was performed at postnatal day 65 (PD65, *n* = 3), the peak of tumour growth. Body weight of ovariectomized females was measured daily from PD65 to PD79 for 14 days and plotted relative to each mouse's baseline (initial weight). Among Cre+ females (*n* = 5), only one survived until PD79. The number of surviving mice at each time point is indicated in the graph.

FST288 (200 μg/kg) was administered twice daily from PD65 to PD78 (*n* = 10 per group) [33, 34, 35]. Solvent with 20‐mM HEPES (pH 7.5) and 500‐mM NaCl was injected to the control group (Cre− and Cre+). Body weight was measured daily from PD65 to PD81 and plotted relative to each mouse's baseline (initial weight).

All animal procedures were approved by UNMC's Institutional Animal Care and Use Committee and followed relevant guidelines. Mice had ad libitum access to food and water and were housed under controlled temperature, humidity and a 10/14‐h light/dark cycle in UNMC's Comparative Medicine facilities.

### 2.2 Measurement of Food Intake

Food intake for ovariectomized Cre−/Ovx and Cre+/Ovx mice was measured from PD65 to PD79 and calculated as the average daily intake per mouse (g/day) during the 2‐week postsurgery until euthanasia. Food intake for Cre− and FST288‐treated Cre+/FST288 mice was measured from PD70 to PD79 and calculated as average daily intake per mouse (g/day). When two mice shared a cage, total intake was divided by two to estimate individual consumption. Mice were cohoused by genotype, and food intake was recorded daily each morning [10].

### 2.3 Adipocyte Size Measurement

Adipocyte size was measured by analysing images of haematoxylin and eosin (H&E)‐stained adipose tissue sections using Adiposoft 1.16 in Manual Model. Three adipose tissue sections per mouse were analysed. About 300 adipocytes per mouse were measured for an average diameter.

### 2.4 Ex Vivo Pancreas Culture

Whole pancreatic organ culture was performed following the established procedure [9, 36]. The pancreas collected from adult C57BL/6 mice were isolated and cultured on 0.4‐μm millicell standing cell culture inserts (PICM01250, MilliporeSigma, Burlington, MA) with a culture medium composed of Minimum Essential Medium‐α (α‐MEM, 32571036, Gibco, Waltham, MA) supplemented with 1 mg/mL bovine serum albumin (BSA, BP9703100, Thermo Fisher Scientific, Waltham, MA). The pancreas was treated with 0‐ or 10‐nM activin A or 20‐nM FST288 and harvested at 48 h and fixed using Modified Davison's Fixative (3600, EKI, Joliet, IL) and sectioned at 5‐μm thickness.

### 2.5 Tissue Harvest and H&E Staining

At euthanasia, pancreas, muscles (tibialis anterior, quadriceps and gastocnemius), adipose tissues (gonadal and subcutaneous), kidney and spleen were fixed in Modified Davidson's Fixative (3600, EKI), sectioned at 5 μm and stained with H&E. Acinar cells, units and cytoplasmic areas were quantified using ImageJ.

### 2.6 Cell Culture

Acinar 266‐6 (CRL‐2151, ATCC) cell was cultured in RPMI1640 (31800022, Gibco) supplemented with 10% foetal bovine serum (FBS, 10082147, Gibco) and 1% antibiotic‐antimycotic (15240062, Gibco) under a 5% CO_2_ incubator at 37°C. The cell line was used at the passages between 8 and 13 and was treated with 0, 1 pM, 1‐ or 10‐nM activin A, or 20‐nM FST288.

### 2.7 Immunofluorescence (IF) Assays and DAB (3, 3‐Diaminobenzidine) Staining

IF assay and DAB staining were performed following established procedures [37, 38]. The Metal Enhanced DAB Substrate Kit (34 065, Thermo Fisher Scientific) was used for DAB staining, and the Alexa Fluor 488 Tyramide SuperBoost Kit (B40932, Invitrogen, Waltham, MA) was used for IF staining. The catalogue numbers and dilutions of primary antibodies were as follows: Insulin (3014, 1:100), α‐Smooth Muscle Actin (α‐SMA) (D4K9N) (19 245, 1:100), phospho‐SMAD3 (Ser423/425) (C25A9) (9520, 1:100), phospho‐Histone H3 (Ser10) (9701, 1:100), SMAD3 (C67H9) (9523, 1:50) and Glycogen Synthase 1 (GYS1) (3893, 1:50) from Cell Signaling Technology (Danvers, MA); Amylase (PA5‐25330) from Invitrogen; Activin Receptor Type IIB (ACVR2B) (ab128544, 1:50) from Abcam (Cambridge, United Kingdom); and Collagen Type IV (AB8201, 1:50) from MilliporeSigma. Amylase signal intensity was quantified using ImageJ from at least five random, nonoverlapping images per sample under identical exposure settings across four experiments. Images were captured with the EVOS M7000 Imaging System (Invitrogen).

### 2.8 Immunocytohistochemistry

Acinar 266‐6 cells were plated on slides, fixed with 4% paraformaldehyde for 10 min at room temperature, permeabilized with 0.1% Triton X‐100 for 10 min, blocked for 60 min and incubated with primary antibody for 1 h. Afterward, secondary antibody was applied for 1 h, followed by 4′,6‐diamidino‐2‐phenylindole (DAPI) staining and mounting.

### 2.9 Immunoblotting

Immunoblotting was performed following established procedures [37]. Briefly, pancreases were harvested and immediately flash‐frozen in liquid nitrogen. Samples were homogenized in RIPA lysis and extraction buffer (89 900, Thermo Fisher Scientific) supplemented with 1× protease and phosphatase inhibitor cocktail (PPC1010, MilliporeSigma). Proteins were loaded onto the protein gel and transferred to a nitrocellulose membrane using the Trans‐Blot Turbo Transfer System (1704150, Bio‐Rad Laboratories Inc., Hercules, CA). The blots were incubated with primary antibodies, followed by secondary antibodies. The catalogue numbers and dilutions of primary antibodies were as follows: SMAD3 (C67H9) (9523, 1:50) and GAPDH (D16H11) XP (5174, 1:1000) from Cell Signaling Technology, Amylase (PA5‐25330) from Invitrogen and pS423/pS425 SMAD3 (600‐401‐919, 1:100) from Rockland Immunochemicals Inc. (Pottstown, PA). The blots were then visualized using the iBright CL1500 Imaging System (A44114, Invitrogen). Protein quantification was performed using ImageJ.

### 2.10 Picro‐Sirius Red Staining

Collagen I and III fibres were visualized using the Picro‐Sirius Red (PSR) Stain Kit (Mercedes Scientific, Lakewood Ranch, FL) on tissue sections (*n* = 3), following the manufacturer's protocol and established methods. Fibrosis was quantified as Collagen Proportionate Area, the ratio of PSR‐stained collagen to total tissue area, analysed at 40X magnification. Quantification in Fiji was repeated three times to ensure reproducibility.

### 2.11 Trichrome Staining

Fibrosis was assessed using Masson's Trichrome Stain Kit (25088, Polysciences, Warrington, PA) on tissue sections (*n* = 3) per the manufacturer's protocol. Slides were deparaffinized, treated with Bouin's fixative and stained sequentially with Weigert's Iron Hematoxylin, Biebrich Scarlet‐Acid Fuchsin solution, Phosphomolybdic‐Phosphotungstic Acid solution and Aniline Blue solution, 1% acetic acid, dehydration, and then mounted.

### 2.12 Terminal Deoxynucleotidyl Transferase dUTP Nick End Labelling (TUNEL) Assay

TUNEL staining was performed using the DeadEnd Fluorometric TUNEL System (G3250, Promega, Madison, WI) and the TUNEL Assay Kit‐HRP‐DAB (ab206386, Abcam), following the manufacturer's instructions.

### 2.13 Measurement of Serum Amylase and Tissue Amylase Activity

Serum amylase activity was measured using a standard curve generated with purified α‐amylase. A 1% starch solution in 50‐mM phosphate buffer (pH 7.0) was prewarmed to 37°C. Serum (50 μL) was mixed with 500 μL of starch solution and incubated at 37°C for 10 min. For tissue amylase activity, 50–100 mg of tissue was homogenized on ice and centrifuged (12 000×*g*, 10 min, 4°C), and the supernatant was used for analysis. Tissue extract (50 μL) was incubated with starch solution (500 μL) at 37°C for 10 min. Iodine reagent (200 μL) was then added, and absorbance was measured at 660 nm. Reduced absorbance reflects starch degradation by amylase.

### 2.14 Toluidine Blue 0O (TBO) Staining

Pancreatic tissue slides were immersed in 1% w/v Toluidine Blue Stain Solution (14949, EKI) diluted in 5% NaCl for 5 min, rinsed thoroughly, dehydrated, cleared with xylene and mounted with a coverslip.

### 2.15 Statistical Analysis

Graphs were generated using Prism 10.5.0 (GraphPad Software, CA) and are shown as mean ± SEM. Normality and variance homogeneity were tested. Outliers and descriptive statistics were assessed via multivariable analysis. Independent *t* tests with Mann–Whitney tests were used for two‐group comparisons; repeated‐measure ANOVA with Type III sums of squares was used for comparisons across multiple groups. Sample sizes were based on prior studies to ensure 80% power at a 0.05 significance level and are indicated in each figure. *p* < 0.05 was considered statistically significant. ns, *, **, *** and **** indicate not significant, *p* < 0.05, *p* < 0.01, *p* < 0.001 and *p* < 0.0001, respectively.

## 3 Results

### 3.1 Acinar Cell Atrophy and Pancreatic Damage in GCT Mice During Cachexia

**FIGURE 1:**
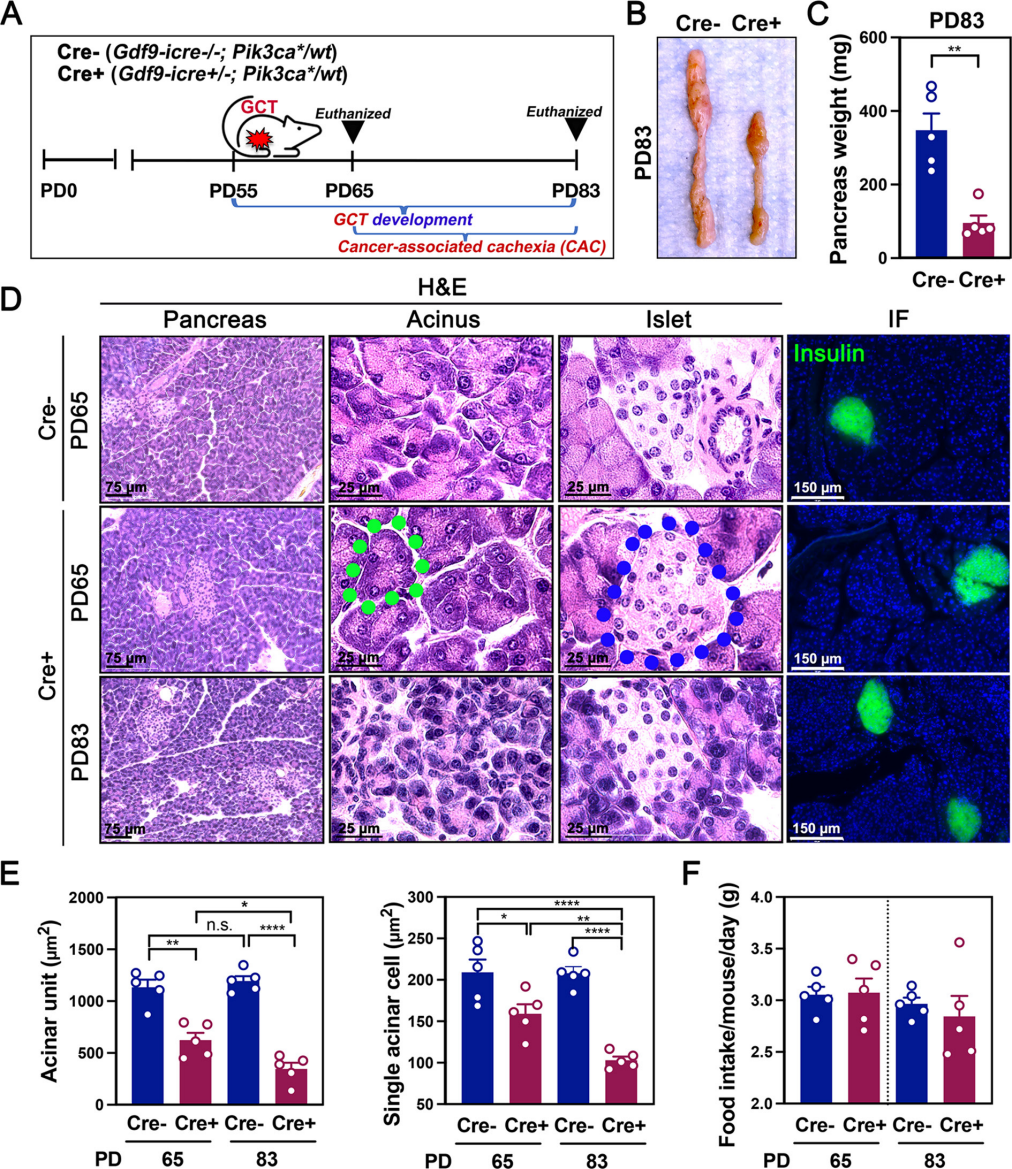
Pancreatic damage in GCT mice during GCT and CAC development. (A) Schematic timeline illustrating the experimental design. *Gdf9‐icre−/−*; *Pik3ca* ^*/^ *wt* and *Gdf9‐icre+/−*; *Pik3ca* ^*/^ *wt* transgenic mice were monitored for GCT and CAC development from Postnatal Day 0 (PD0) to PD83. (B) Representative images of pancreas from Cre− (*n* = 5) and Cre+ (*n* = 5) mice at PD83. (C) Pancreas weights at PD83 in Cre− and Cre+ mice. (D) Representative H&E‐stained pancreatic tissue sections. Left panels show overall tissue morphology; middle panels highlight acinar cell architecture, with a representative acinar unit outlined in green. Right panels display islet regions, with representative islets outlined in blue. Immunofluorescence (IF) images show insulin‐positive cells (green) and nuclei counterstained with DAPI (blue). Pancreatic H&E images were captured using a 20× objective, and all other images were captured using a 40× objective. (E) Quantification of acinar unit area and single acinar cell size. (F) Daily food intake per mouse measured in Cre− and Cre+ mice at PD65 and PD83.

To investigate pancreas changes during skeletal muscle atrophy and adipose tissue wasting at the onset of CAC, pancreatic phenotypes were examined in the GCT mouse model previously shown to develop CAC [10, 30, 31]. Bilateral GCTs appeared around PD55–PD60 in Cre+ mice and grew to large tumour sizes, leading to progressive body weight loss from PD70 to PD83 [10, 30, 31]. Mice were euthanized at PD65 or PD83 to assess pancreatic alterations (Figure 1A). Representative images and quantitative analysis revealed significantly reduced pancreatic size and weight in Cre+ mice at PD83, indicating marked atrophy (Figures 1B,C and S1A). H&E staining further demonstrated progressive acinar cell atrophy and pancreatic damage at both time points in Cre+ mice (Figure 1D). At higher magnification, acinar cells in Cre+ mice at PD83 appeared smaller, with reduced cytoplasmic volume, disorganized architecture and condensed nuclei, indicating cellular degeneration (Figure 1D). In contrast, pancreatic islets remained structurally intact, and insulin staining confirmed preserved β‐cell populations, suggesting maintained islet function (Figure 1D). Morphometric analyses revealed significant reductions in total acinar area, individual cell size and cytoplasmic volume, particularly at PD83 (Figures 1E and S1B). Despite pronounced pancreatic damage, food intake at PD65 and PD83 did not differ between groups (Figure 1F), indicating that digestive function may remain unaffected during tumour progression.

### 3.2 Pancreatic Dysfunction and Skeletal Muscle Atrophy in CAC

**FIGURE 2:**
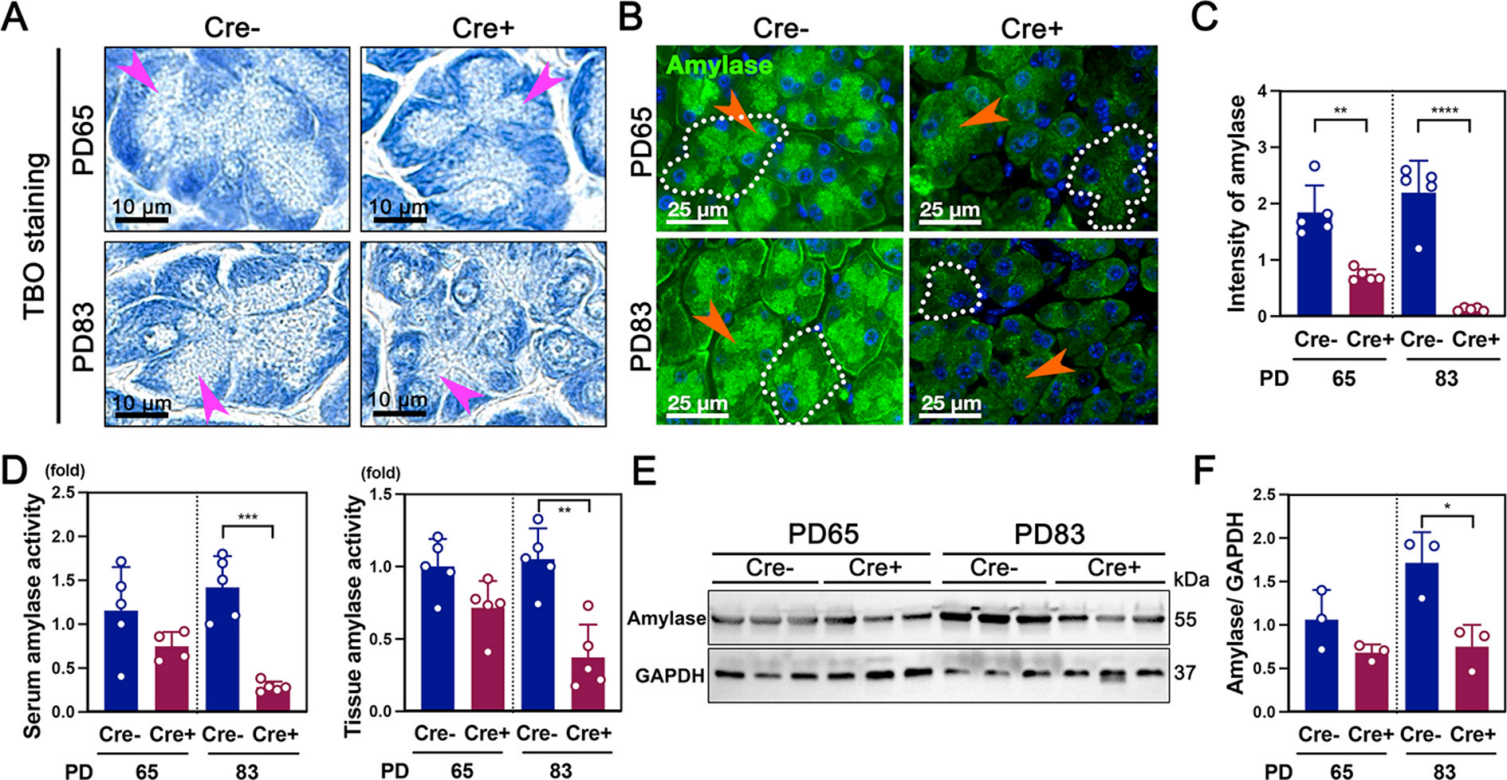
Pancreatic acinar cell function in GCT mice. (A) Toluidine Blue O (TBO) staining of pancreatic tissues from Cre− (*n* = 5) and Cre+ (*n* = 5) mice at PD65 and PD83. Pink arrowheads indicate the area rich in zymogen granules. Images were captured using a 40× objective. (B) IF staining of amylase in pancreatic sections from Cre− and Cre+ mice at PD65 and PD83. Orange arrows highlight amylase expression in Cre− and Cre+ mice. Dotted circles outline representative acinar units. Images were captured using a 40× objective. (C) Quantification of amylase fluorescence intensity. (D) Serum and tissue amylase activity assays. (E, F) Western blot analysis and quantification of amylase protein levels in pancreatic tissue at PD65 and PD83.

To investigate the impact of cachexia on pancreatic acinar cells in the GCT‐induced cachexia mouse model, Toluidine Blue O (TBO) staining was performed to visualize both nuclei and cytoplasm. TBO staining revealed a marked a reduction in acinar cell size in Cre+ mice compared to Cre− controls, particularly at PD83 (Figure 2A). Correspondingly, amylase, a key exocrine pancreatic enzyme, was significantly decreased in Cre+ mice, as demonstrated by immunofluorescence staining (Figure 2B). Quantification of amylase fluorescence confirmed a significant reduction at PD83 (Figure 2C). Additionally, amylase enzymatic activity in both serum and pancreatic tissue was significantly lower in Cre+ mice at PD83, further supporting exocrine dysfunction (Figure 2D). Western blot analysis of amylase protein levels corroborated these findings, showing a marked decrease in Cre+ mice at PD83 (Figure 2E,F), confirming exocrine pancreatic insufficiency. Cachexia also leads to skeletal muscle loss, contributing to weakness and metabolic decline. H&E staining of muscle tissues revealed significantly smaller muscle fibres in the tibialis anterior (TA), quadriceps (Q) and gastrocnemius (G) of Cre+ mice at PD83, indicating pronounced muscle atrophy (Figure S2A). Immunostaining for glycogen synthase (GYS) showed reduced glycogen content in Cre+ mice, with decreased fluorescence intensity at both PD65 and PD83 (Figure S2B), reflecting impaired glucose metabolism and energy depletion during cachexia progression.

### 3.3 Pancreatic Fibrosis and Acinar Cell Death in the Pancreas During Cachexia

**FIGURE 3:**
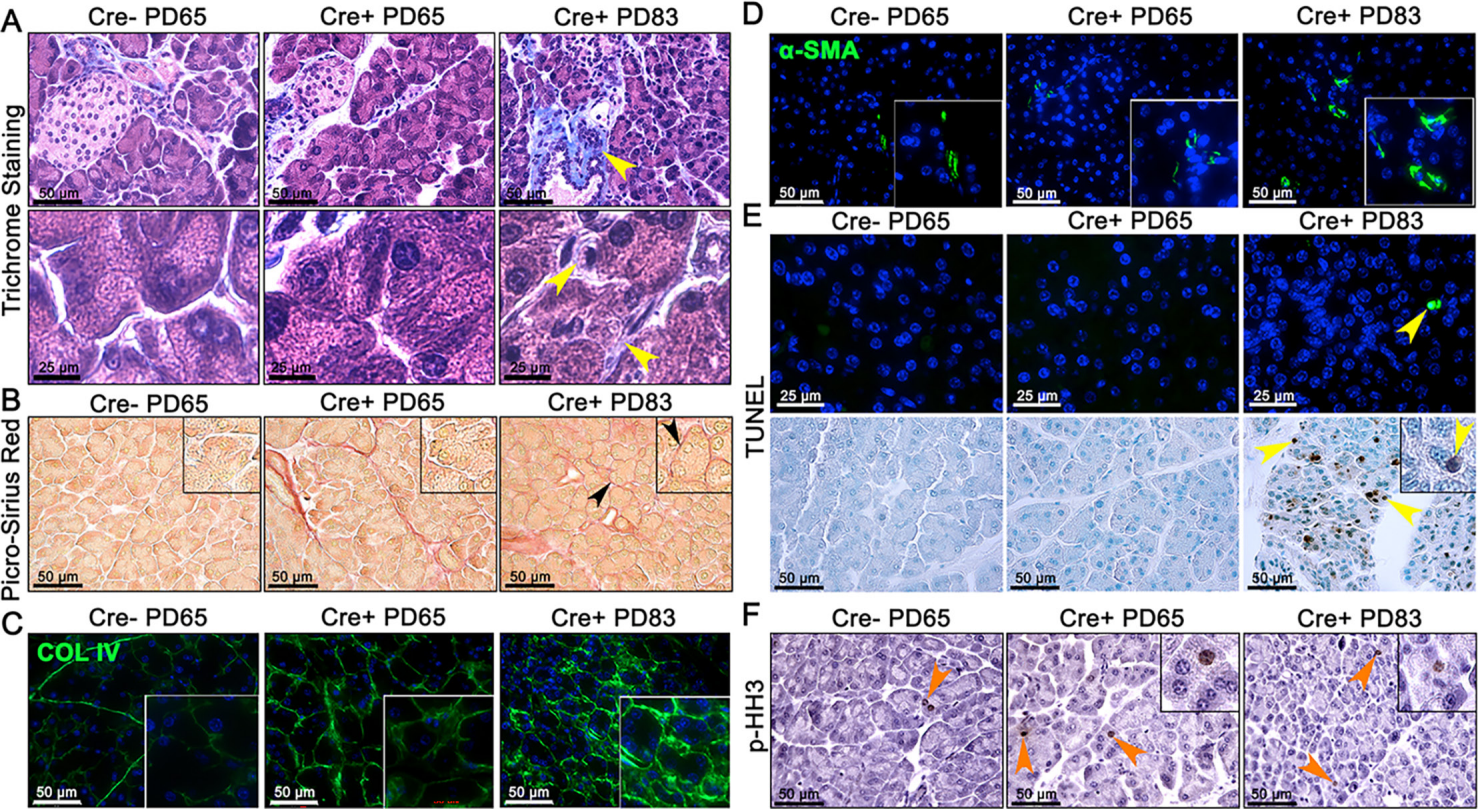
Fibrosis, tissue remodelling and apoptosis in acinar cells during cachexia progression. (A) Representative images of Trichrome staining showing collagen deposition (blue staining indicated with yellow arrowheads) in pancreatic tissue sections (*n* = 4 per group). (B) Picro‐Sirius Red staining highlights collagen fibres (black arrowheads) (*n* = 3 per group). Insets show magnified views of acinar cells. (C) Assessment of basement membrane integrity using collagen IV (COL IV) immunostaining (*n* = 3–6 per group). (D) IF staining of α‐SMA (*n* = 4 or 5 per group). Insets show magnified views of α‐SMA‐positive cells. (E) TUNEL assay to detect apoptotic acinar cells using IF (top) and DAB (bottom) staining (*n* = 4 per group). TUNEL‐positive cells are indicated by yellow arrowheads. (F) DAB staining of phospho‐histone H3 (p‐HH3) to identify mitotic cells (orange arrowheads) (*n* = 3 per group). Insets provide magnified views of p‐HH3‐positive cells. Images were captured using a 40× objective.

To investigate the mechanism underlying pancreatic damage during cachexia progression, histological and molecular analyses were performed to assess fibrosis, apoptosis and cellular proliferation. Trichrome staining was used to evaluate collagen deposition and fibrotic changes. At PD65, both Cre− and Cre+ groups exhibited normal pancreatic architecture with well‐organized acinar structures and minimal collagen deposition, as indicated by the absence of trichrome staining (Figure 3A). In contrast, Cre+ mice at PD83 displayed pronounced structural disruption and increased collagen accumulation, with blue‐stained fibrotic regions surrounding atrophied acinar units (yellow arrowheads, Figure 3A), indicating a significant fibrotic response.

Picro‐Sirius Red staining further confirmed the presence of fibrosis, revealing collagen fibre deposition, likely Types I and III, around the shrunken acini in Cre+ PD83 samples (black arrowheads, Figures 3B and S3A). These findings suggest that unknown factors promote extracellular matrix remodelling and fibrosis during cachexia. Immunostaining for collagen IV and α‐SMA in Cre+ PD83 pancreas revealed elevated expression (Figures 3C,D and S3B,C), indicative of basement membrane disruption and activation of a fibrotic microenvironment that may compromise tissue integrity and regenerative capacity.

Given the disorganized tissue architecture, we next assessed cellular turnover. TUNEL staining revealed increased apoptosis of acinar cells in Cre+ PD83 mice, with numerous TUNEL‐positive cells (yellow arrowheads, Figures 3E and S3D), whereas Cre− PD65 and Cre+ PD65 samples showed no apoptotic signals. In contrast, phosphorylated histone H3 (p‐HH3) immunostaining showed little to no mitotic activity in acinar cells across all groups (Figures 3F and S3E), suggesting that cell death rather than reduced proliferation contributes to acinar cell loss. These results indicate that cachexia progression leads to pancreatic fibrosis and acinar cell death, contributing to impaired organ function.

### 3.4 GCTs as a Causal Factor for CAC

To determine whether GCTs directly contribute to the development of CAC, including body weight loss, adipocyte depletion and pancreatic dysfunction, ovariectomy was performed at PD65, a time point characterized by large GCTs and preceding the onset of cachexia in Cre+ mice (Figure S4A). As demonstrated in our previous study [10], Cre+ mice exhibited progressive weight loss indicative of cachexia‐induced metabolic deterioration. Notably, ovariectomy in Cre+ mice prevented severe weight loss, suggesting that tumour‐driven ovarian factors contribute to systemic wasting (Figure S4B), despite comparable food intake between Cre+/Ovx and Cre−/Ovx mice (Figure S4C). Adipose tissue mass analysis showed significantly reduced gonadal and subcutaneous adipose tissue in Cre+ mice, whereas ovariectomy preserved adipose tissue stores (Figure S4D), indicating that ovarian GCTs promote lipid mobilization. Consistently, Cre+ mice exhibited marked pancreatic damage, as evidenced by reduced pancreatic weight, whereas ovariectomy restored pancreas mass (Figure S4E). Histological analysis revealed disorganized and atrophic acinar structures in Cre+ mice, whereas ovariectomy preserved acinar morphology (Figure S4F, top row). Immunofluorescence staining for amylase demonstrated a significant reduction in Cre+ mice, whereas ovariectomy restored amylase expression, suggesting that GCTs and their secreted factors contribute to pancreatic dysfunction (Figure S4F, bottom row).

### 3.5 Activin A Signalling and Acinar Cell Morphology

**FIGURE 4:**
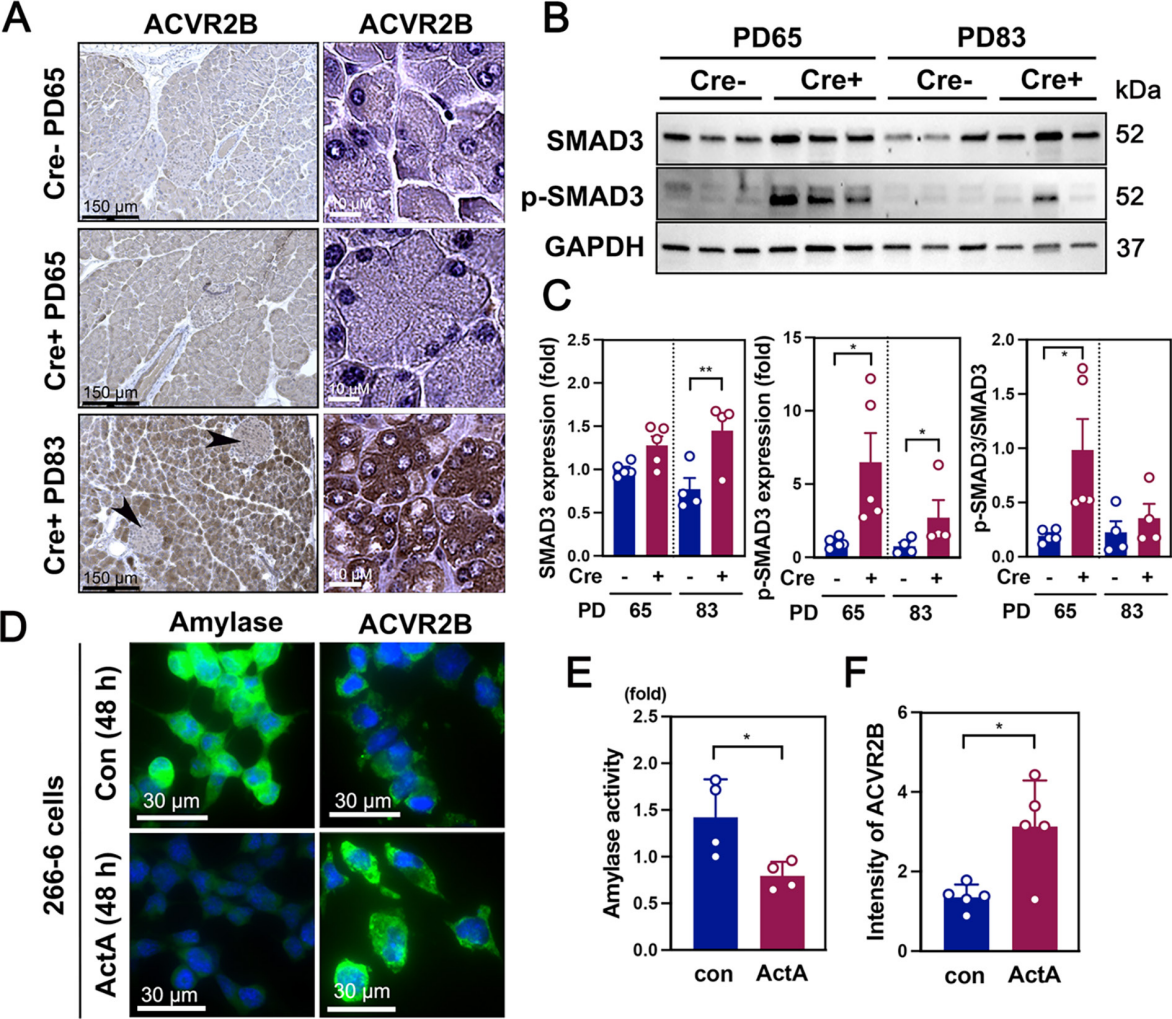
Activin A signalling contributes to acinar cell atrophy. (A) DAB staining of ACVR2B in pancreatic tissues from Cre− PD65, Cre+ PD65 and Cre+ PD83 mice. Left panels show low magnification, and right panels show high magnification. A 20× objective was used for ACVR2B images in the left panel, and a 40× objective was used for ACVR2B images in the right panel. (B) Western blot analysis of total SMAD3 and phospho‐SMAD3 (p‐SMAD3) in pancreatic tissue from Cre− and Cre+ mice at PD65 and PD83. GAPDH was used as a loading control. (C) Quantification of western blot signals for SMAD3, p‐SMAD3 and p‐SMAD3/SMAD3 ratio. (D) Immunocytohistochemistry of amylase and ACVR2B in 266‐6 pancreatic acinar cells after 48 h of control or activin A (ActA) treatment. Images were captured using a 40× objective. (E) Amylase activity in 266‐6 cells under control and ActA treatment for 48 h. (F) Quantification of ACVR2B expression intensity in 266‐6 cells following 48‐h control or ActA treatment.

Given the elevated levels of activin A observed in GCT Cre+ mice during CAC development, we investigated its mechanism of action on acinar cells. DAB staining revealed that ACVR2B, a receptor for activin A, was highly expressed in pancreatic acinar cells but exhibited low expression in islet cells (Figure 4A, black arrowheads), indicating that activin A selectively affects acinar cells. Immunofluorescence confirmed increased nuclear p‐SMAD3 in acinar cells of Cre+ PD65 mice compared to Cre− PD65 and Cre+ PD83 mice (Figure S5A). Western blot analysis further demonstrated significantly increased SMAD3 phosphorylation in pancreatic tissues from Cre+ PD65 mice (Figure 4B,C), indicating activation of downstream signalling by activin A prior to acinar cell atrophy at PD83. Ex vivo pancreatic tissues treated with activin A (ActA) for 48 h exhibited pronounced morphological changes, as shown by H&E and amylase staining (Figure S5B). Quantification revealed a significant reduction in both acinar cell size and total acinar area (Figure S5C), supporting a direct impact of activin A on acinar structure. Amylase activity in ex vivo tissues declined markedly following activin A treatment, decreasing from baseline at 0 h to very low levels by 48 h (Figure S5D), indicating impaired acinar function. Similarly, activin A treatment of 266‐6 acinar cells led to a dose‐dependent reduction in amylase activity (Figure S5E). Amylase expression and activity decreased (Figure 4D,E), whereas ACVR2B expression increased substantially after activin A exposure (Figure 4D,F).

### 3.6 Activin A in CAC and the Protective Effects of FST288

**FIGURE 5:**
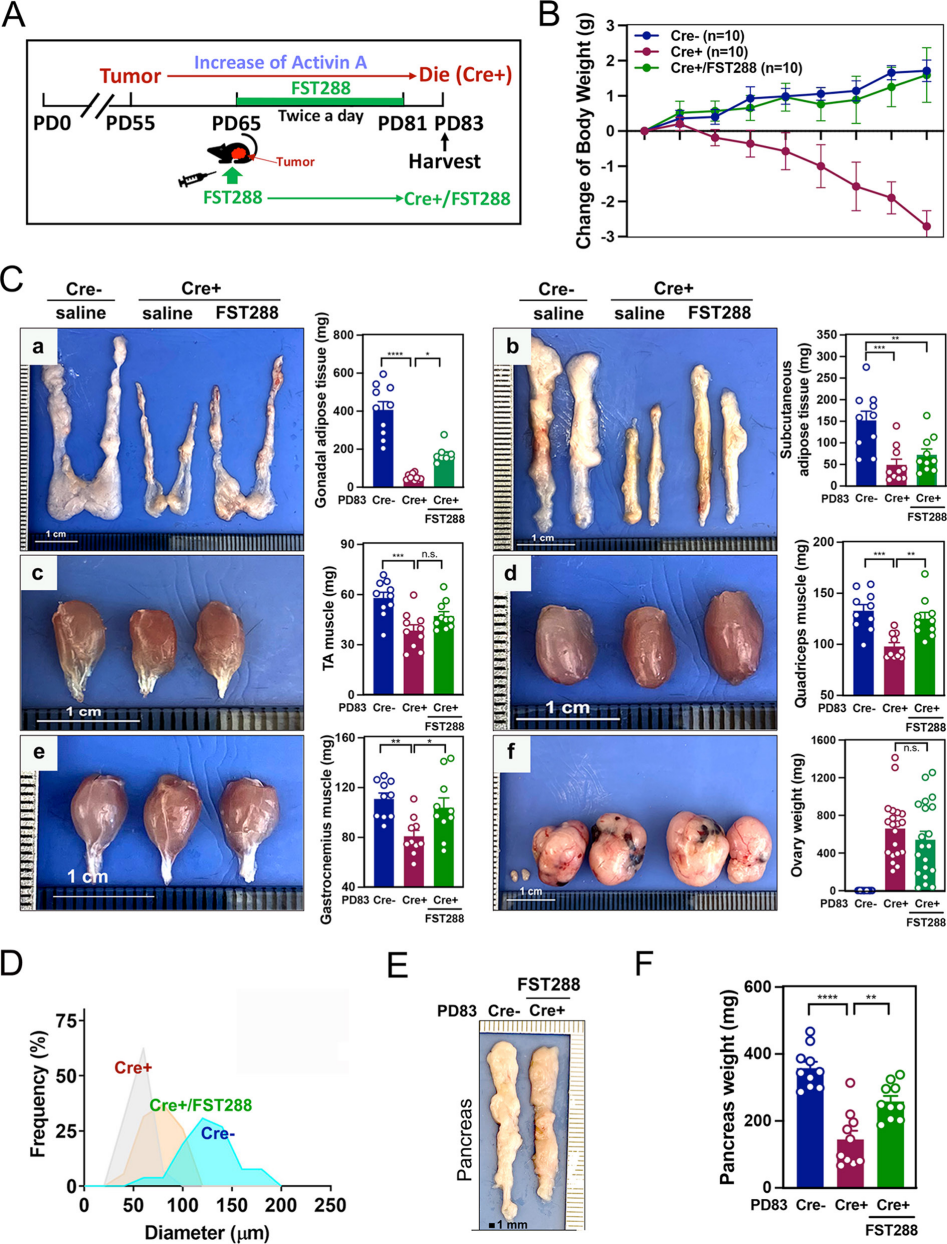
FST288 treatment mitigates CAC symptoms in PD83 Cre+ mice. (A) Schematic of the experimental timeline. FST288 treatment was administered twice daily from PD65 to PD81. (B) Change of body weight over time. (C) Representative images and quantification of gonadal adipose tissue (a), subcutaneous adipose tissue (b), TA muscle (c), quadriceps muscle (d), gastrocnemius muscle (e) and ovary (f) from Cre−, Cre+ and Cre+/FST288 groups. (D) Frequency distribution of adipocyte diameter in gonadal adipose tissue. (E) Representative images of pancreatic tissues. (F) Pancreatic weight.

To determine whether FST288, an activin A antagonist, could counteract the deleterious effects of elevated activin A signalling, FST288 was administered twice daily for 2 weeks in Cre+ tumour‐bearing mice (Figure 5A). Food intake remained comparable across all groups, indicating that the weight loss observed in Cre+ mice was due to metabolic dysregulation rather than reduced consumption (Figure S6A,B). Notably, body weight significantly increased in Cre+/FST288 mice compared to untreated Cre+ mice, demonstrating that FST288 mitigated GCT‐driven cachexia‐induced wasting (Figure 5B). Adipose and skeletal muscle tissues, key indicators of cachexia‐related metabolic imbalance, were also assessed. Cre+/FST288 mice exhibited significantly increased gonadal and subcutaneous adipose tissues as well as larger tibialis anterior (TA), quadriceps (Q) and gastrocnemius (GC) muscles, consistent with improved body weight (panels a–e in Figures 5C and S6C–F). Importantly, tumour size and remained unaffected by FST288 treatment (panel f in Figure 5C). The histology of GCTs revealed no differences between solvent‐ and FST288‐treated tumours, with tumour cells present in both groups (Figure S6G). Picro‐Sirius Red staining revealed diminished collagen deposition in the gonadal and subcutaneous adipose tissues of Cre+/FST288 mice, closely resembling the pattern observed in Cre− controls, suggesting that FST288 alleviates cachexia‐associated fibrosis (Figure S6E). Analysis of adipocyte size distribution showed that the majority of gonadal adipocytes in Cre+/FST288 mice measured 50–100 μm, compared to 30‐ to 70‐μm Cre+ mice (Figure 5D). Most notably, pancreatic weight was partially restored following FST288 treatment (Figure 5E,F), indicating a protective effect on pancreatic integrity and function.

### 3.7 Rescue of Activin A–Induced Pancreatic Damage by FST288

**FIGURE 6:**
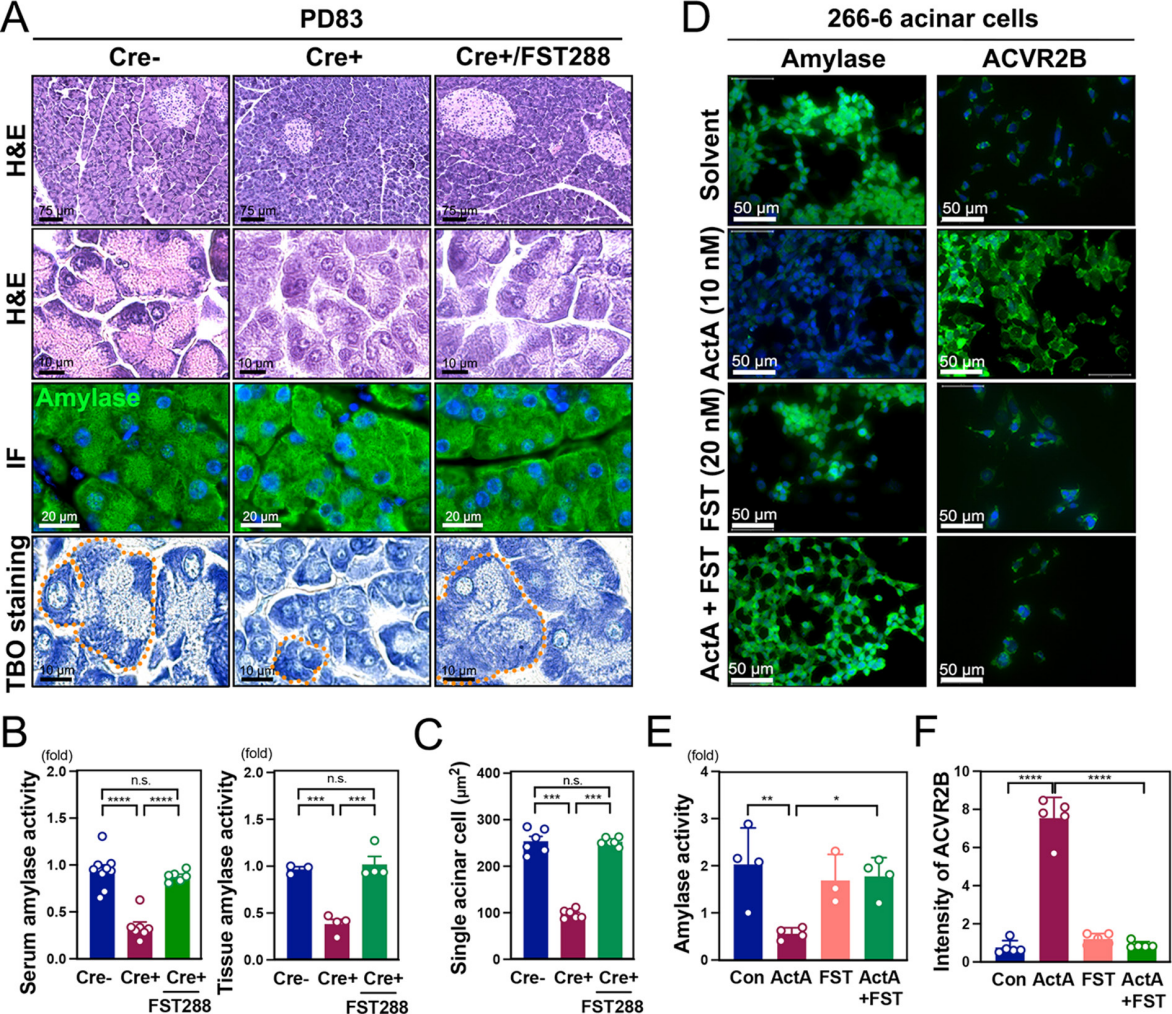
FST288 rescues pancreatic acinar atrophy induced by activin A. (A) Histological and IF analysis of pancreatic tissue from PD83 Cre−, Cre+ and Cre+/FST288 mice. Upper panels: H&E staining; middle panels: IF staining of amylase; lower panels: Toluidine Blue O staining. Dotted circles outline representative acinar units. The H&E images in the top panel were acquired with a 20× objective, whereas the remaining images were captured with a 40× objective. (B) Quantification of serum and tissue amylase activity. (C) Measurement of individual acinar cell area across groups. (D) IF staining of amylase and ACVR2B in 266‐6 pancreatic acinar cells treated with solvent, ActA, FST288 or ActA + FST288 (ActA + FST). Images were captured using a 40× objective. (E) Quantification of amylase activity in 266‐6 cells under the indicated treatments. (F) Quantification of ACVR2B expression in 266‐6 cells under the indicated treatments.

To evaluate the protective effects of FST288 on acinar cells, we examined its impact on pancreatic morphology and function. H&E and immunofluorescence staining revealed disrupted acinar architecture and reduced amylase expression in Cre+ mice, whereas FST288 treatment preserved acinar morphology and restored amylase levels (Figure 6A). Toluidine Blue O (TBO) staining further confirmed preservation of acinar cell structure in Cre+/FST288 mice (Figure 6A). Insulin expression remained unchanged across groups following FST288 administration, indicating no significant impact on islet cells (Figure S7A). Biochemical analyses demonstrated that FST288 significantly restored both serum and tissue amylase activity (Figure 6B) as well as acinar cell size (Figure 6C), underscoring its protective role. In ex vivo pancreatic tissue cultures, activin A markedly reduced acinar size and amylase expression (Figure S7B), whereas cotreatment with FST288 effectively blocked these effects and preserved acinar integrity. Quantitative measurements confirmed that FST288 prevented activin A–induced damage at both the single‐cell and acinar unit levels (Figure S7C). Correspondingly, amylase activity was significantly restored by FST288 cotreatment (Figure S7D). Consistent results were observed in 266‐6 pancreatic acinar cells. Activin A reduced amylase expression, whereas FST288 reversed this suppression (Figure 6D). Quantitative analysis further confirmed that FST288 significantly mitigated activin A‐induced reductions in amylase activity and ACVR2B expression (Figure 6E,F).

## 4 Discussion

This study demonstrates that GCT progression and the subsequent onset of cachexia result in significant pancreatic damage in GCT mice, primarily affecting the exocrine pancreas. GCT Cre+ mice exhibit a loss of acinar cell mass, reduced cytoplasmic volume of acinar cells, a decrease in zymogen granules and diminished amylase expression and enzymatic activity, ultimately disrupting pancreatic homeostasis. Compared to acinar cell atrophy, insulin‐producing β‐cells remain intact, as evidenced by histologically normal islets and sustained insulin expression, suggesting that exocrine tissue is more sensitive to systemic cachexia. Additionally, GCT Cre+ mice exhibit glycogen depletion in accordance with severe skeletal muscle atrophy, indicative of cancer‐associated cachexia.

GCT Cre+ mice exhibited progressive pancreatic fibrosis characterized by extensive collagen deposition and basement membrane remodelling. Despite the severity of fibrosis, apoptosis levels remained relatively low, suggesting that remodelling may occur through a nonapoptotic mechanism. Alternatively, apoptosis may proceed gradually, contributing to pancreatic dysfunction over time. Acinar cells can undergo dedifferentiation or transdifferentiation into progenitor‐like phenotypes through activation of stress‐ and inflammation‐associated signalling pathways, thereby promoting fibrosis, loss of function and tumourigenesis. Consequently, acinar cell plasticity may serve as a central mechanism driving pancreatic fibrosis and metabolic decline in GCT‐induced cachexia.

Activin A, a member of the TGF‐β superfamily, promotes fibrosis by activating SMAD‐dependent signalling in fibroblasts, thereby inducing collagen deposition and extracellular matrix remodelling [39]. This fibroblast activation enhances the synthesis and secretion of extracellular matrix components, including collagen. Additionally, activin A can induce epithelial‐to‐mesenchymal transition (EMT), in which epithelial cells transform into myofibroblasts, contributing to fibrosis in organs such as the lung, liver and heart [40]. In this study, GCT‐derived activin A likely drives pancreatic fibrosis and acinar cell dysfunction. Blocking activin A with FST288 appears to reduce fibrosis in the pancreas.

GCT induced a marked loss of acinar cell mass, characterized by reduced cytoplasmic volume, fewer zymogen granules and diminished amylase expression and activity. Remarkably, ovariectomy alleviated these effects, leading to weight gain, improved activity, healthy fur and extended survival. Additionally, it alleviated cachexia‐induced weight loss, adipose tissue depletion and pancreatic damage in GCT Cre+ mice, suggesting that tumour‐derived ovarian factors drive systemic wasting and organ dysfunction. These findings suggest that eliminating the source of cachexia‐inducing factors can reverse cachexia symptoms, thereby improving overall health and survival. Specifically, activin A produced during GCT growth appears to be a key mediator of pancreatic damage.

Although our GCT mouse model does not fully replicate the age‐related incidence or gradual onset characteristic of human patients, it recapitulates critical clinical features, including progressive weight loss, skeletal muscle atrophy and adipose tissue depletion. It serves as a robust system for studying CAC‐related metabolic dysfunction and tissue damage. During disease progression, serum levels of activin A and GDF15 rose dramatically (76‐ and 10‐fold, respectively), alongside elevated IL‐6 and TNF‐α [10]. In this study, FST288 was administered for 2 weeks beginning at PD65, when GCTs were established and secreting activin A. By PD83, untreated Cre+ mice showed marked loss of skeletal muscle and adipose tissue mass. In contrast, FST288‐treated Cre+ mice maintained muscle and adipose tissue mass, with steady weight gain. Adipocyte size was restored to levels comparable to healthy controls, indicating improved adipose integrity. FST288 also preserved pancreatic mass and acinar structure, significantly improving serum and tissue amylase activity. These results suggest that FST288 not only prevents pancreatic damage but also supports digestive function during cachexia. Follistatin exhibits broader antagonistic effects beyond activin A, including inhibition of GDF11 and myostatin [41], which may contribute to the outcomes observed in this study. Our data do not determine whether the rescue of pancreatic function by follistatin represents a primary mechanism or a secondary effect mediated through its ability to restore muscle mass. However, given follistatin's capacity to modulate multiple pathways, it is plausible that improvements in muscle mass, as well as other downstream effects mediated by GDF11 and myostatin inhibition, collectively contribute to the physiological benefits observed. These possibilities should be considered when interpreting the findings and underscore the need for future studies to dissect the relative contributions of these pathways. Overall, this study identifies FST288 as a promising therapeutic approach for protecting against CAC‐induced tissue wasting and metabolic impairment, with potential to preserve pancreatic enzyme function and counteract muscle degradation in cachectic conditions.

Interestingly, GCT size did not decrease with FST288 administration, as tumour sizes between Cre+ and Cre+/FST288 mice remained comparable. This suggests that GCT growth is not solely dependent on activin A. Alternatively, it is possible that the timing or dosage of FST288 treatment did not align with the critical phase of tumour growth, thereby failing to suppress key tumour‐driving events and permitting continued tumour progression. However, FST288 effectively mitigated the effects of secreted activin A on other organs, helping to ameliorate severe consequences and side effects associated with cachexia. The underlying mechanisms driving GCT growth require further investigation to better understand how to modulate these processes for therapeutic benefit.

In this study, we demonstrated that SMAD3 signalling is highly active in acinar cell nuclei and that activin A induces acinar cell atrophy using ex vivo pancreatic tissue and 266‐6 cell cultures. Blocking activin A reversed atrophy and restored amylase expression. However, the exact mechanism by which activin A‐SMAD3 signalling causes pancreatic damage and reduces amylase remains unclear. Additionally, the study did not examine other digestive enzymes (proteases, lipases and nucleases) or the regulation of their expression in acinar cells. Although we confirmed histologically intact pancreatic islets and the presence of insulin expression, the functional activity of insulin (β‐cells), glucagon (α‐cells), somatostatin (δ‐cells), pancreatic polypeptide (PP‐cells or γ‐cells) and ghrelin (ε‐cells) was not assessed. Future studies should focus on elucidating the endocrine–pancreatic interactions that contribute to pancreatic damage and recovery, particularly in the context of metabolic dysfunction. A deeper understanding of these pathways may provide valuable insights into therapeutic strategies to mitigate pancreatic damage, preserve metabolic function and ultimately improve the quality of life for cancer patients.

## Ethics Statement

The authors affirm compliance with the ethical guidelines of the *Journal of Cachexia, Sarcopenia and Muscle*.

## Conflicts of Interest

The authors declare no conflicts of interest.

## Supporting information


**Figure S1:** Pancreatic damage in GCT Mice. **(A)** Pancreas weight normalized to body weight at PD83 in Cre‐ and Cre + mice. **(B)** Cytoplasmic area of individual acinar cells at PD65 and PD83 in Cre‐ and Cre + mice.

**Figure S2:** Skeletal muscle atrophy in GCT mice. **(A)** Analysis of tibialis anterior (TA), quadriceps (Q), and gastrocnemius (G) muscles in Cre‐ and Cre + mice at PD65 and PD83. Representative H&E staining (top) and quantification (bottom). Images were captured using a 40 × objective. **(B)** Glycogen synthase (GYS) IF staining (top) and quantification (bottom). Quantification of integrated density for GYS IF staining in skeletal muscles in Cre‐ and Cre + mice at PD65 and PD83.

**Figure S3:** Quantification of signals in PD65 Cre‐, PD65 Cre+, and PD83 Cre+. (A) Picro‐Sirius Red‐positive area (*n* = 3). (B) Area of Collagenase IV expression (*n* = 3–6). (C) Area of α‐SMA expression (*n* = 4–5). (D) TUNEL‐positive cells (*n* = 4 per group). (E) p‐HH3‐positive cells (*n* = 3 per group).

**Figure S4:** Ovariectomy mitigates CAC and pancreatic damage in GCT Cre+ mice. **(A)** Experimental timeline showing tumour development and the timing of ovariectomy (Ovx). GCTs developed in Cre + mice around PD55, leading to CAC. Ovx was performed at PD65, and mice were harvested at PD79 for analysis. **(B)** Body weight was recorded every other day starting on the day of surgery for Cre−/ovariectomy (*n* = 3), Cre + (*n* = 5), and Cre+/ovariectomy (*n* = 3) mice. For Cre + mice, body weight changes of surviving individuals are shown. **(C)** Daily food intake per mouse from PD65 to PD79 in Cre−/ovariectomy (*n* = 3) and Cre+/ovariectomy (*n* = 3) groups. **(D)** Gonadal and subcutaneous adipose tissue mass at PD79. **(E)** Pancreas weight at PD79. **(F)** Histological analysis of pancreatic tissue. H&E staining (top row) and IF of amylase (bottom row) in Cre‐/Ovx at PD65, Cre + at PD79, and Cre+/Ovx mice at PD79. Dotted circles outline representative acinar units. Images were captured using a 40 × objective.

**Figure S5:** Activin A signalling in pancreatic damage. **(A)** IF staining for p‐SMAD3 in pancreatic sections from Cre‐ PD65, Cre + PD65, and Cre + PD83 mice. Nuclei were counterstained with DAPI (blue). **(B)** Ex vivo pancreatic tissue culture treated with activin A (ActA). Representative H&E and amylase IF staining are shown for control (Con) at 0 h and 48 h, and ActA‐treated samples at 48 h. Images were captured using a 40 × objective. **(C)** Quantification of single acinar cell size and acinar unit area in control at 0 h and 48 h and ActA‐treated samples at 48 h. **(D)** Quantification of amylase activity in ex vivo cultured pancreatic tissue. **(E)** Amylase activity in 266–6 pancreatic acinar cells treated with increasing concentrations of activin A (1 pM, 1 nM, and 10 nM).

**Figure S6:** Effects of FST288 treatment on CAC symptoms in PD83 Cre+ mice. **(**A‐B) Daily food intake per mouse measured for 10 days. **(C)** Representative histological images of tibialis anterior (TA), quadriceps (Q), and gastrocnemius (G) muscle. Images were captured using a 40x objective. **(D)** Quantification of TA, Q, and G muscle fibres. CSA, cross‐sectional area. **(E)** Representative histological and Picro‐Sirius Red‐stained images of gonadal and subcutaneous adipose tissues from Cre‐, Cre+, and Cre+/FST288 mice. Images were captured using a 40× objective. (**F**) Measurement of adipocyte diameter in gonadal and subcutaneous adipose tissues from Cre‐, Cre+, and Cre+/FST288 mice. **(G)** Representative histological images of ovarian tumour tissue sections from Cre‐, Cre+, and Cre+/FST288 groups. Images were captured using 10 × and 40 × objectives.

**Figure S7:** Effect of FST288 on rescuing pancreatic damage. **(A)** H&E staining and DAB immunostaining for Insulin in pancreatic acinar cells from PD83 Cre‐, Cre+, and Cre+/FST288 groups. **(B)** Ex vivo analysis of pancreatic tissues treated with solvent, ActA (10 nM), FST288 (20 nM), or a combination of ActA and FST288. Representative H&E and amylase IF staining are shown. Dotted circles outline representative acinar units. Images were captured using a 40 × objective. **(C)** Quantification of single acinar cell and acinus area. **(D)** Amylase activity assay.
