# Supplementary figures and images for: Pancreatic Damage in Ovarian Cancer–Associated Cachexia Is Driven by Activin A Signalling
Source: J Cachexia Sarcopenia Muscle. 2025 Oct 10;16(5):e70096. doi: 10.1002/jcsm.70096 (PMC12512902; doi:10.1002/jcsm.70096)

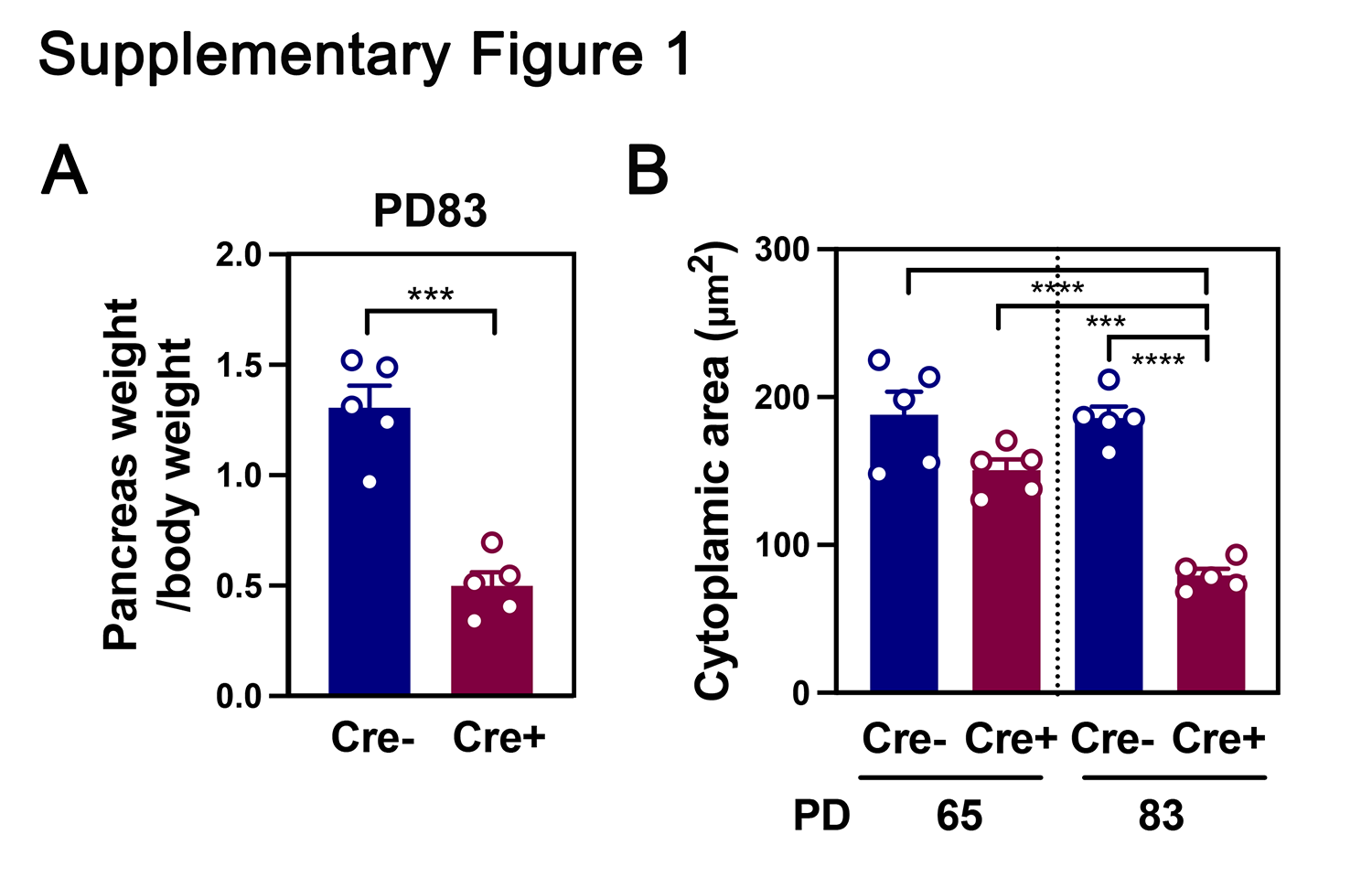

Supplement: Supplementary file 1 — Figure S1: Pancreatic damage in GCT Mice. (A) Pancreas weight normalized to body weight at PD83 in Cre‐ and Cre + mice. (B) Cytoplasmic area of individual acinar cells at PD65 and PD83 in Cre‐ and Cre + mice. [file JCSM-16-e70096-s004.tif]

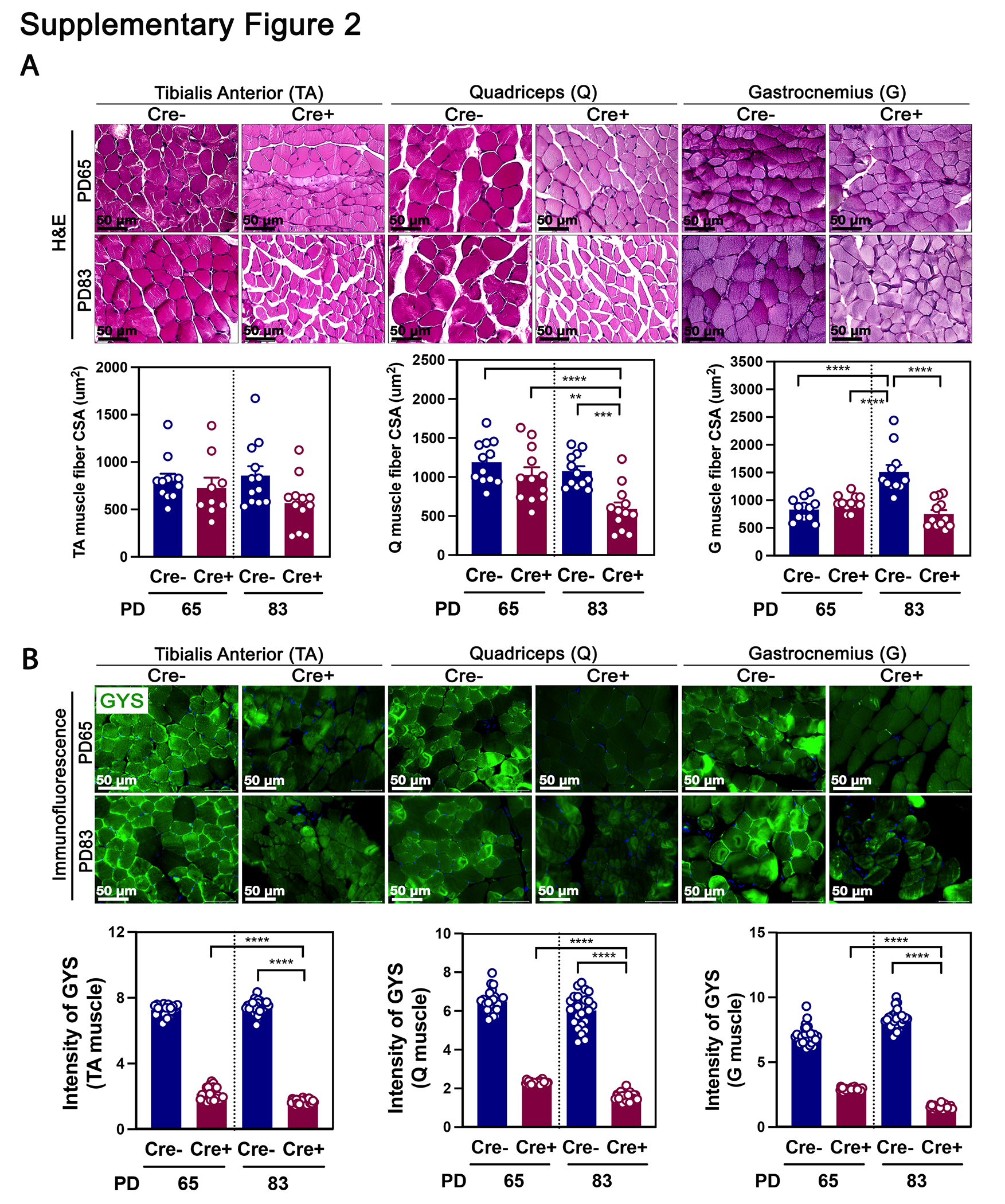

Supplement: Supplementary file 2 — Figure S2: Skeletal muscle atrophy in GCT mice. (A) Analysis of tibialis anterior (TA), quadriceps (Q), and gastrocnemius (G) muscles in Cre‐ and Cre + mice at PD65 and PD83. Representative H&E staining (top) and quantification (bottom). Images were captured using a 40 × objective. (B) Glycogen synthase (GYS) IF staining (top) and quantification (bottom). Quantification of integrated density for GYS IF staining in skeletal muscles in Cre‐ and Cre + mice at PD65 and PD83. [file JCSM-16-e70096-s005.tif]

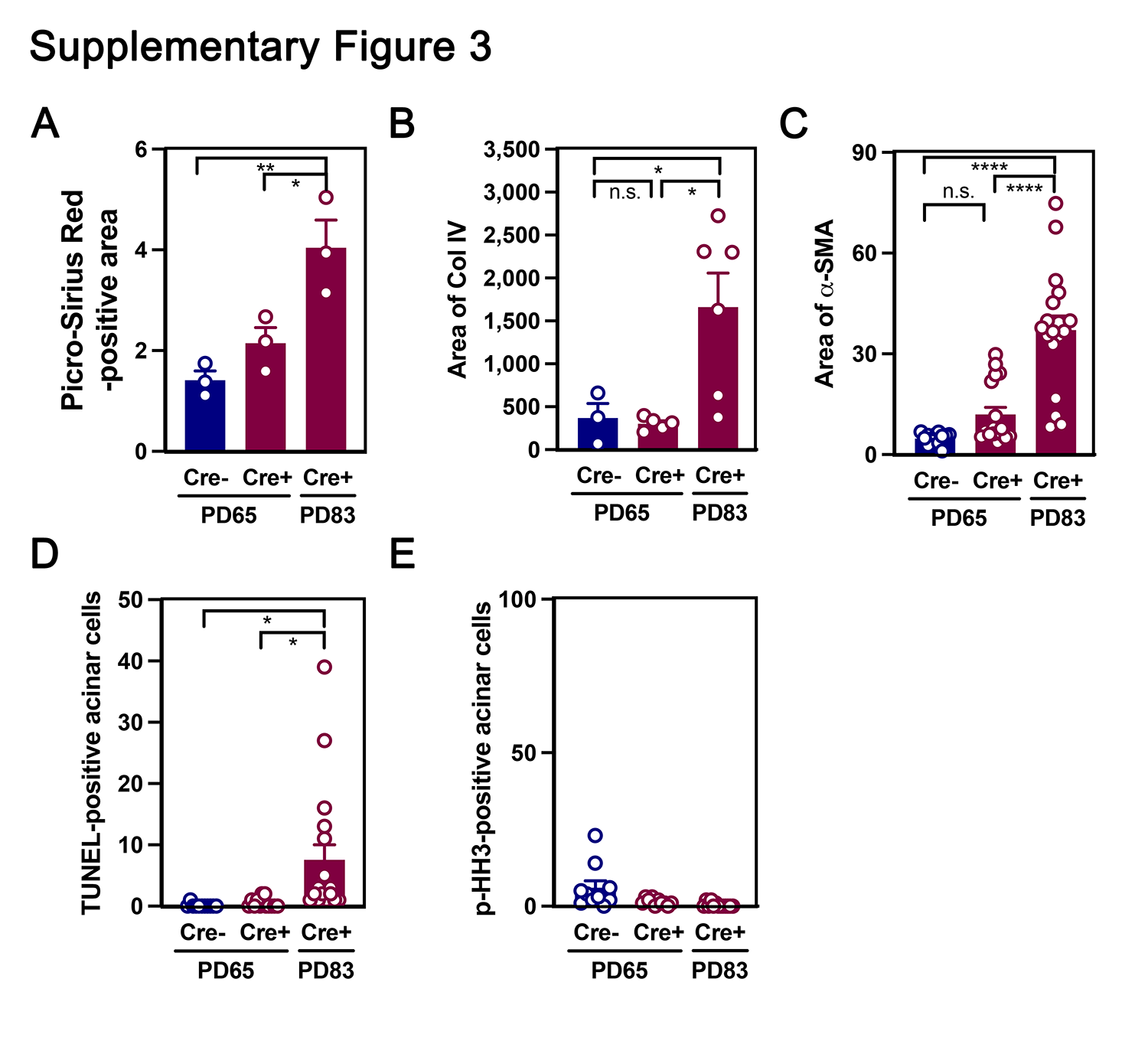

Supplement: Supplementary file 3 — Figure S3: Quantification of signals in PD65 Cre‐, PD65 Cre+, and PD83 Cre+. (A) Picro‐Sirius Red‐positive area (n = 3). (B) Area of Collagenase IV expression (n = 3–6). (C) Area of α‐SMA expression (n = 4–5). (D) TUNEL‐positive cells (n = 4 per group). (E) p‐HH3‐positive cells (n = 3 per group). [file JCSM-16-e70096-s007.tif]

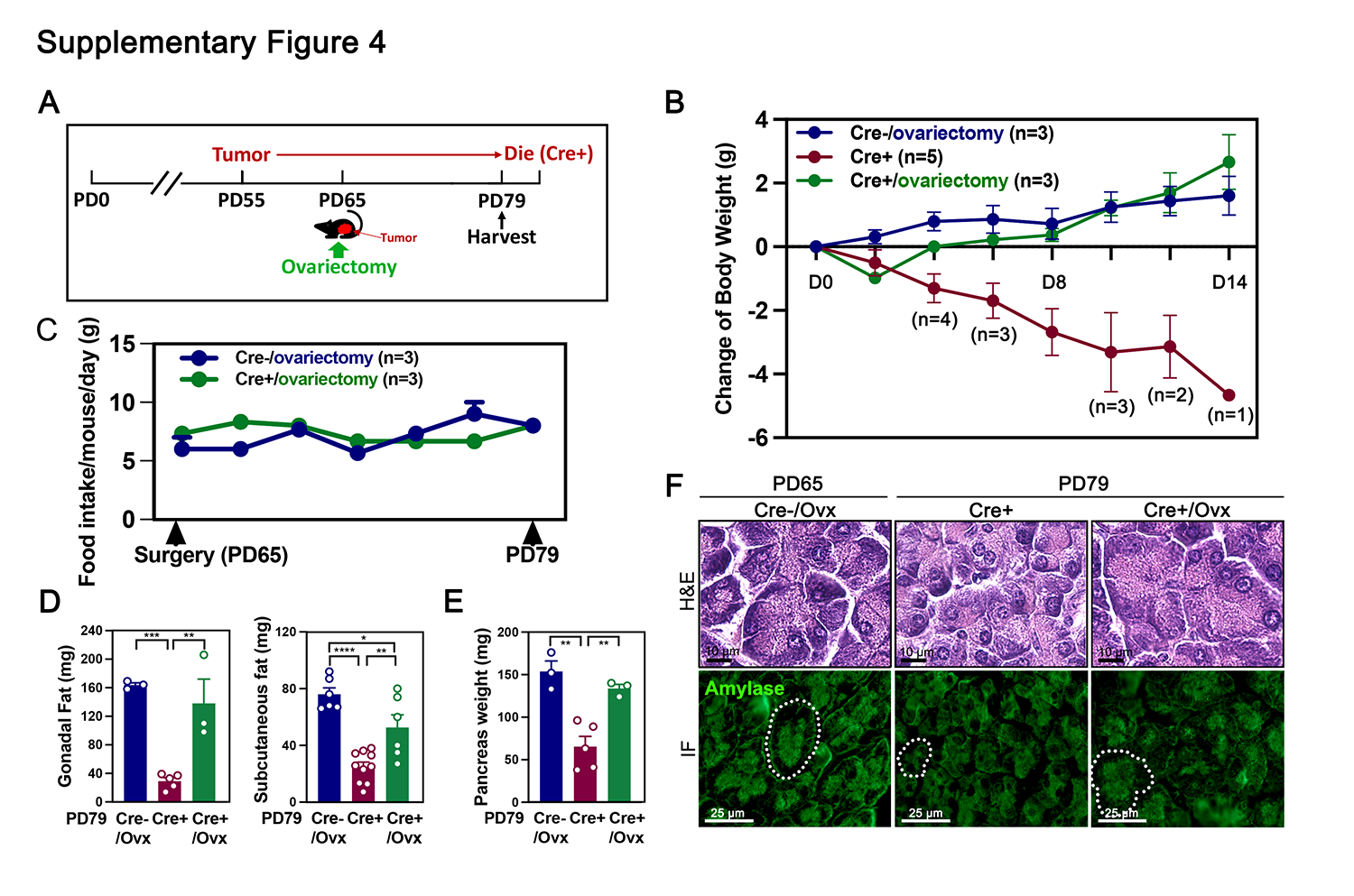

Supplement: Supplementary file 4 — Figure S4: Ovariectomy mitigates CAC and pancreatic damage in GCT Cre+ mice. (A) Experimental timeline showing tumour development and the timing of ovariectomy (Ovx). GCTs developed in Cre + mice around PD55, leading to CAC. Ovx was performed at PD65, and mice were harvested at PD79 for analysis. (B) Body weight was recorded every other day starting on the day of surgery for Cre−/ovariectomy (n = 3), Cre + (n = 5), and Cre+/ovariectomy (n = 3) mice. For Cre + mice, body weight changes of surviving individuals are shown. (C) Daily food intake per mouse from PD65 to PD79 in Cre−/ovariectomy (n = 3) and Cre+/ovariectomy (n = 3) groups. (D) Gonadal and subcutaneous adipose tissue mass at PD79. (E) Pancreas weight at PD79. (F) Histological analysis of pancreatic tissue. H&E staining (top row) and IF of amylase (bottom row) in Cre‐/Ovx at PD65, Cre + at PD79, and Cre+/Ovx mice at PD79. Dotted circles outline representative acinar units. Images were captured using a 40 × objective. [file JCSM-16-e70096-s003.tif]

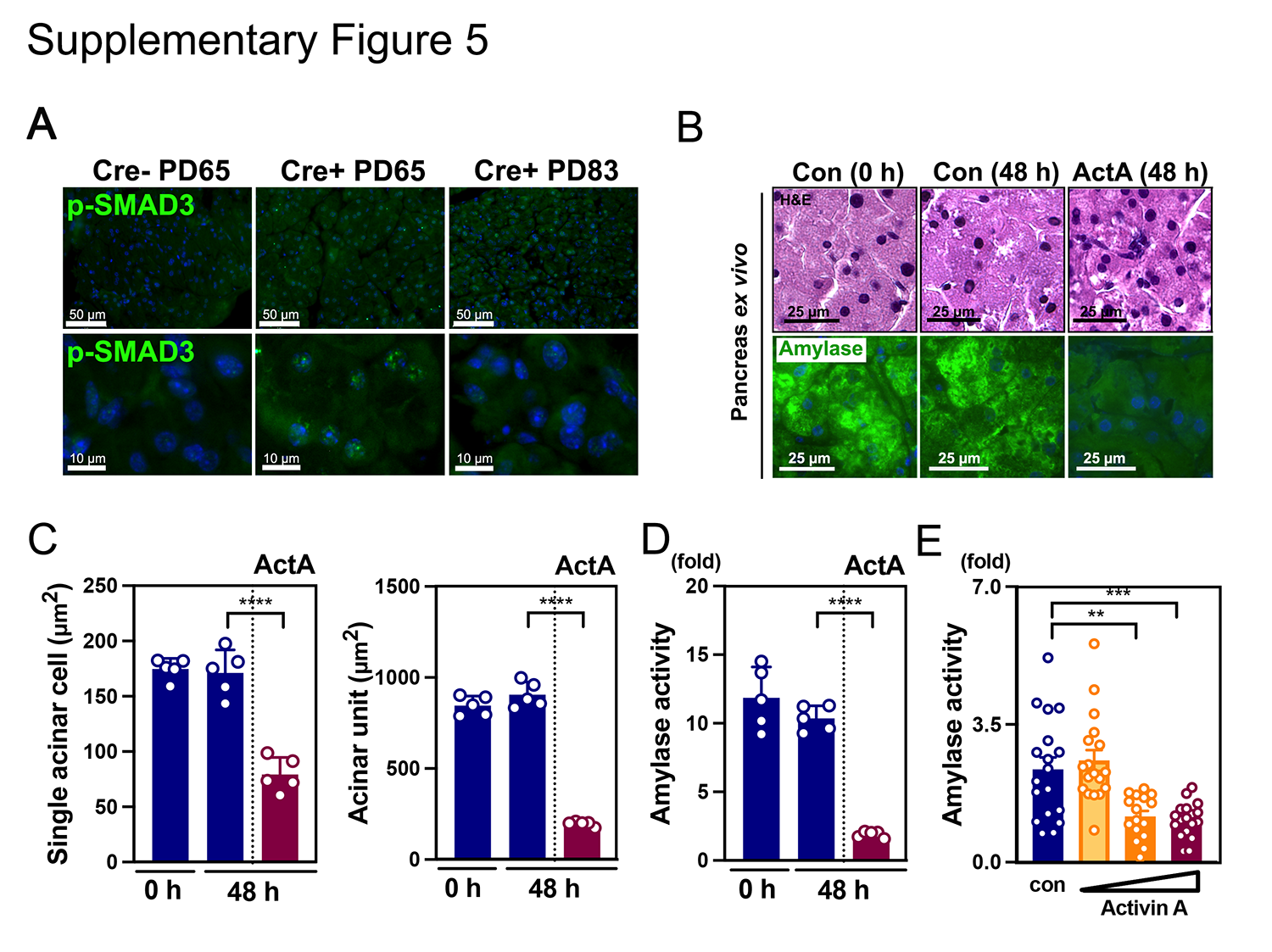

Supplement: Supplementary file 5 — Figure S5: Activin A signalling in pancreatic damage. (A) IF staining for p‐SMAD3 in pancreatic sections from Cre‐ PD65, Cre + PD65, and Cre + PD83 mice. Nuclei were counterstained with DAPI (blue). (B) Ex vivo pancreatic tissue culture treated with activin A (ActA). Representative H&E and amylase IF staining are shown for control (Con) at 0 h and 48 h, and ActA‐treated samples at 48 h. Images were captured using a 40 × objective. (C) Quantification of single acinar cell size and acinar unit area in control at 0 h and 48 h and ActA‐treated samples at 48 h. (D) Quantification of amylase activity in ex vivo cultured pancreatic tissue. (E) Amylase activity in 266–6 pancreatic acinar cells treated with increasing concentrations of activin A (1 pM, 1 nM, and 10 nM). [file JCSM-16-e70096-s006.tif]

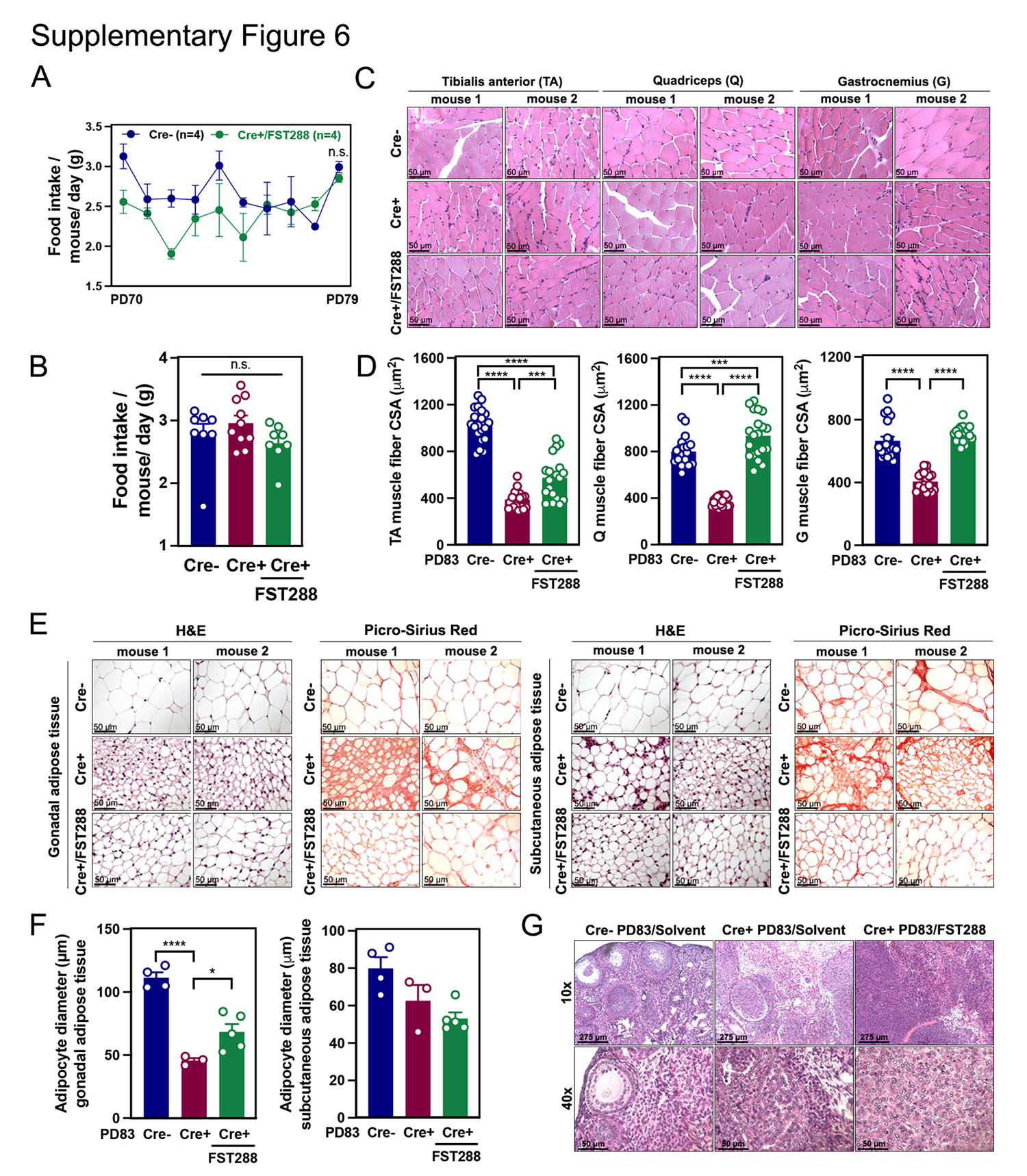

Supplement: Supplementary file 6 — Figure S6: Effects of FST288 treatment on CAC symptoms in PD83 Cre+ mice. (A‐B) Daily food intake per mouse measured for 10 days. (C) Representative histological images of tibialis anterior (TA), quadriceps (Q), and gastrocnemius (G) muscle. Images were captured using a 40x objective. (D) Quantification of TA, Q, and G muscle fibres. CSA, cross‐sectional area. (E) Representative histological and Picro‐Sirius Red‐stained images of gonadal and subcutaneous adipose tissues from Cre‐, Cre+, and Cre+/FST288 mice. Images were captured using a 40× objective. (F) Measurement of adipocyte diameter in gonadal and subcutaneous adipose tissues from Cre‐, Cre+, and Cre+/FST288 mice. (G) Representative histological images of ovarian tumour tissue sections from Cre‐, Cre+, and Cre+/FST288 groups. Images were captured using 10 × and 40 × objectives. [file JCSM-16-e70096-s001.tif]

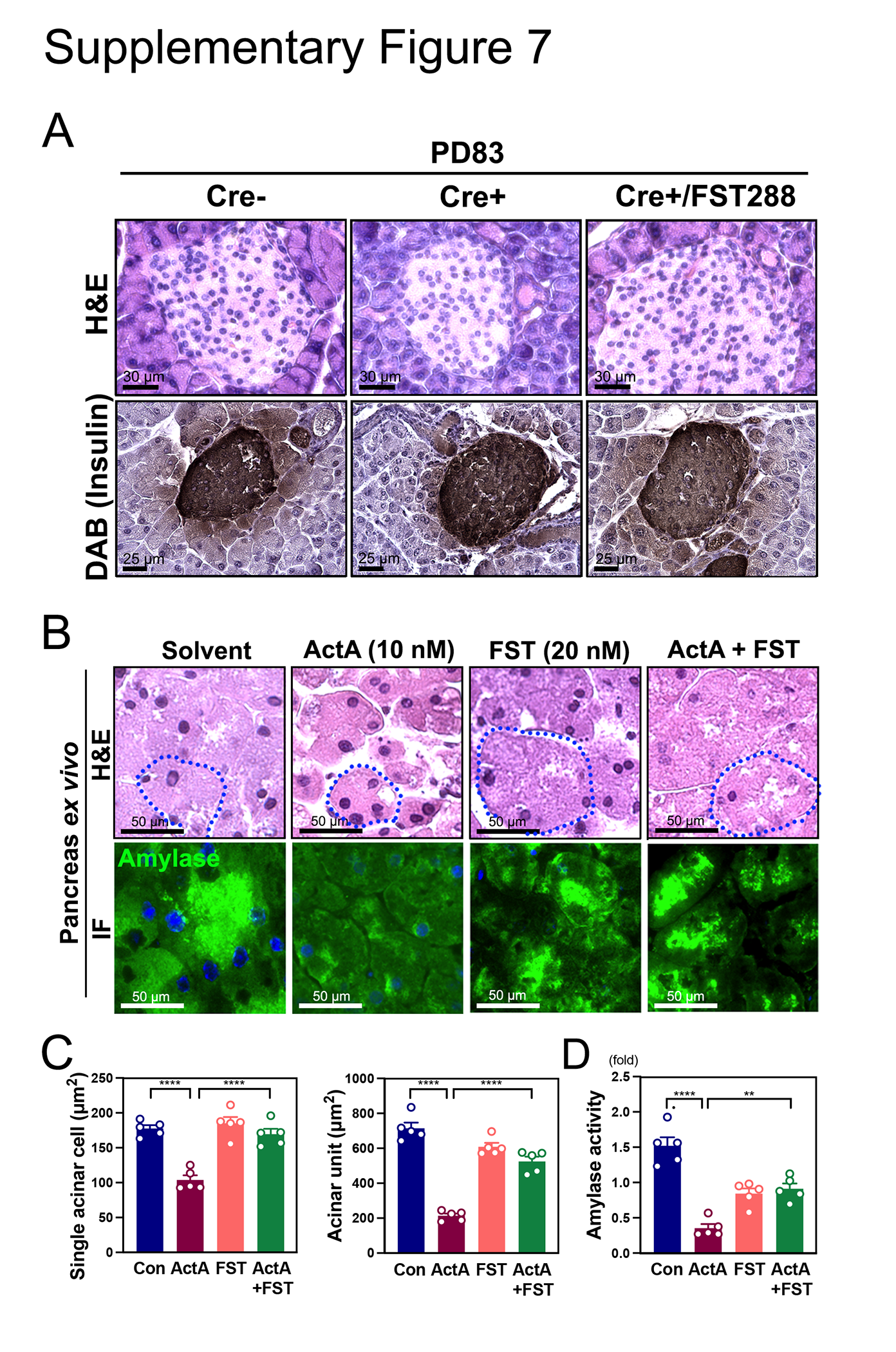

Supplement: Supplementary file 7 — Figure S7: Effect of FST288 on rescuing pancreatic damage. (A) H&E staining and DAB immunostaining for Insulin in pancreatic acinar cells from PD83 Cre‐, Cre+, and Cre+/FST288 groups. (B) Ex vivo analysis of pancreatic tissues treated with solvent, ActA (10 nM), FST288 (20 nM), or a combination of ActA and FST288. Representative H&E and amylase IF staining are shown. Dotted circles outline representative acinar units. Images were captured using a 40 × objective. (C) Quantification of single acinar cell and acinus area. (D) Amylase activity assay. [file JCSM-16-e70096-s002.tif]
